# Mechanisms of cognitive control in cadet pilots

**DOI:** 10.1186/s40696-016-0016-5

**Published:** 2016-03-03

**Authors:** Shirley Gordon, Nir Getter, Idit Oz, Dror Garbi, Doron Todder

**Affiliations:** 1 Psychology Branch, Chief Surgeon Headquarters, IDF Medical Corps, Israeli Air Force, Tel Hashomer, Ramat-Gan, Israel; 2 Mental Health Center, Ministry of Health, Beer Sheva, Israel

**Keywords:** Cadet pilots, Mental rotation test, Dual task, Cognitive control

## Abstract

**Background:**

Optimizing performance of aviators while minimizing risks arising from the exposure to extreme environment, both external and internal, is one of the principles guiding the Israeli Air Force. Young cadets in particular are considered an “at risk” population due to the fact that they have no experience in flight in the first stages of training and are therefore subjects for investigation.

**Methods:**

In this study, we investigated the cognitive performance of young cadet pilots across different hours of the day. 39 cadets were randomly divided into 3 groups: morning, late afternoon, and late evening groups and then tested on a cognitive battery that contained both simple performance measures but also complex measures like dual-tasking and mental rotation test.

**Results:**

The analysis indicated a significant effect of ‘time of day’ on the participants’ accuracy [*F* (2, 32) = 3.4, *p* < 0.05]. In a post hoc pairwise t-tests, we found a near significant (*p* = 0.52) increase in participants’ accuracy and a significant increase [*F* (2, 32) = 4.5, *p* < 0.05] in participants’ reaction time in the late evening group as compared to the morning group. We also found a differential effect of dual tasking on accuracy in the different daytimes [*F* (2, 33) = 5.6, *p* < 0.01]. In a post hoc analysis, we found that accuracy in the 1-back task deteriorates from single task condition to the dual task condition only in the morning group (*p* < 0.05), but not in the late evening or late-afternoon group.

**Conclusions:**

This ‘trade-off’ behavior, slowing down in order to perform better, in the late evening group may be a result of a voluntary control mechanism (top-down processes) activated at night, in this group. The combination of feeling fatigue, along with the understanding that complex tasks are more resource consuming, caused the cadets to check and double-check before answering, whereas in the morning group, they felt alert and vital, and acted more reactively, ended in an impulsive manner that caused to inaccurate performance.

## Background

In the field of medical aviation, most of the research investigates the influence of fatigue on aviator’s performance [4–8, 16, 18, 19, 23, 24] or the influence of psychostimulants, such as modafinil (provigil) on performance in deprived aviators [3, 5]. However, to the best of our knowledge, there are no studies in this field that investigate changes in *cognitive control* processes across the different hours of the day. We find this question to be very important since it has a direct influence on the training programs in the cadet’s academy.

Cognitive control is defined as the ability to organize, monitor and regulate low-level cognitive processes (such as perceptual, motor, and memory processes) in order to match information processing to the current situation’s demands. Braver’s [2] new model of dual mechanisms of control (DMC) is aimed to explain the *variability* in cognitive control processes. The DMC model refers to two distinct modes of control: ‘proactive control’ and ‘reactive control’. The proactive control is when goal-relevant information is actively maintained in a sustained manner until the action is completed whereas in reactive control, attention is recruited in a just in time manner and it is stimulus-driven goal reactivation. The proactive control strategy is strongly resource consuming, requiring continuous goal maintenance, however behavior is continually adjusted to facilitate successful completion of the goal. In other words, less economic but more efficient. By contrast, the reactive control strategy has the advantage of being economically-efficient yet sometimes in a price of inaccuracies.

In this study, our intent was to investigate the cognitive performance of young cadet pilots across different hours of the day (morning, late afternoon and late evening). These young cadets have a very tight and intensive schedule routine, and although they are well selected and have high cognitive skills, at this point in their training, they are *not* highly trained, and have little experience in actual flight. Since flight is an environment of high mental-load, mechanisms of cognitive control are expected to be activated. Moreover, cognitive control mechanisms will be even more pronounced in inexperienced aviators, like cadet-pilots, before automatization processes start to occur as a result of training. Therefore, we hypothesized that these cadet pilots may adopt different cognitive strategies across the day as a result of internal and external environmental cues that may have a direct influence on their aerial functioning. More specifically, in the morning, when they feel alert and vital, they will be more reactive and responsive, whereas in the late hours of the day, as a result of fatigue, they may slow down in their performance. At first glance, high functioning in the morning and decrease in performance in the late hours of the evening may sound trivial. However, we also hypothesize that during high mental load tasks (i.e. flight), performance in the late evening hours of the day will be slower yet more accurate, whereas performance in the early hours of the day will be fast yet less accurate.

Or in other words, we expect that the ‘reactive control’ mode will be activated when these cadets will have an inner feeling of vitality and the ‘proactive control’ mode will be activated when their inner feeling will be of fatigue and not an “at peak” feeling, which is usually occurs during the late hours of the evening [9, 22].

### The present study

The aim of the present study was to investigate whether there are changes in cognitive control across the different hours of the day (morning, late afternoon and late evening). We used a cognitive battery that was designed for that purpose. The cognitive battery contained both simple measures, such as simple reaction time in a psychomotor vigilance task, measures of motor inhibition and complex measures like dual-tasking and mental rotation tests, abilities associated with flying [14]. Our hypothesis was that in this high functioning population, the best performance will be detected in the late afternoon group as compared to the morning or late evening group. We also hypothesized that a decline will be seen *only* in the performance on complex measures for the morning and late evening groups, due to the high mental load of the measures and the high cognitive profile of this specific population.

## Methods

### Participants

In this study we recruited 39 cadet pilots forming one cohort in their academic phase of their military training, aged 21–23, with no history of psychiatric disorders, head trauma, central nervous system disorders or use of psychotropic medications. All participants were right handed, with normal or corrected sight, speak and understand Hebrew fluently, and with 15 years of education. In order to minimize the individual differences that are more related to situational differences (like sleeping hours, time of meals, etc.), we tested a group of cadet pilots that have the same routine, in a group-testing procedure. They all performed the cognitive test-battery simultaneously. All participants were randomly assigned into one-session examination of either morning group (07:00 a.m., *N* = 11), late-afternoon group (06:00 p.m., *N* = 13), or a late evening group (10:00 p.m., *N* = 15)^1^ and were then tested on a cognitive battery that is described in greater detail in the measures section. No significant differences were found between groups in age [*M* = 20.6, *SD* = 0.67 for the morning group, *M* = 21.38, *SD* = 1.32 for the late-afternoon group, *M* = 21.4, *SD* = 1.5 for the late evening group, *F*(2, 36) = 1.42, *ns*], gender [2 female (morning group) vs 2 female (late-afternoon group), vs no female (late evening group), no female (late evening group) *p* = 0.2, by Fisher’s exact test], or hours of sleep in the previous night before the test [*M* = 5.5, *SD* = 0.77 for the morning group, *M* = 6.4, *SD* = 1.01 for the late-afternoon group, *M* = 5.3, *SD* = 1.6 for the late evening group, *F*(2, 36) = 3.017, *p* = 0.06]. There were no differences between the groups in academic achievements or in intelligence scores [*M* = 112, *SD* = 13 for Morning group, *M* = 110, *SD* = 14 for late-afternoon group, *M* = 117, *SD* = 13 for late-night group, *F* (2, 36) = 1.15, *ns*].

All participants were informed that participating in the study is voluntary, and that no personal details about their achievements will be provided to their superiors or have any effect on their professional progress. The Israel Defense Force’s Medical Corps Institutional Review Board approved the study.

### Measures

The experiment was run on Lenovo laptop computers with high-resolution screens of 13–14 inches. The procedures for all tests-battery were programmed in open-sesame [17], which is a freely distributed software. All tasks were automatically initiated at the predefined order by a single graphic user interface that was available to the participant. This approach prevented confusion of switching or skipping tasks and was necessary in a group-testing format. The cognitive battery was assembled from a collection of well-established cognitive tasks, often used in studies of cognitive psychology research.

#### The Cognitive battery

The battery included 6 tasks (in this order): simple reaction time, choice reaction time, go/no-go, anti-saccade, dual task (tracking + 1-Back), and mental rotation test.

A brief description of the tasks follows below.

**Fig. 1:**
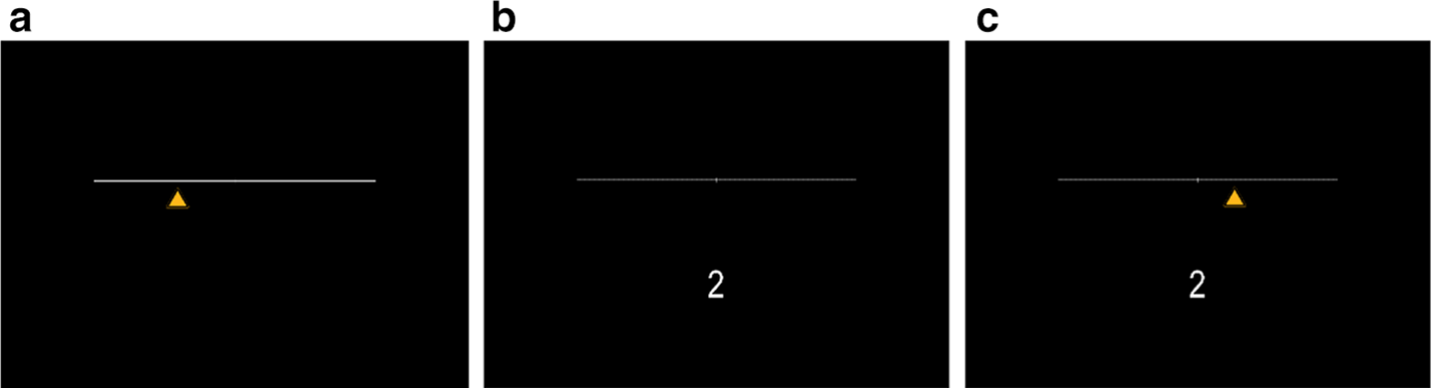
Dual task (visual tracking + 1-back)

**Fig. 2:**
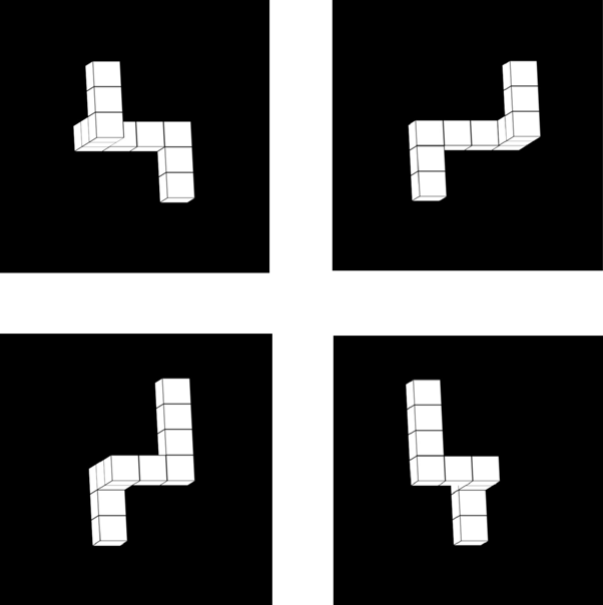
Mental rotation test (MRT)

1. **Simple Reaction Time (SRT):** This task was designed to measure the participant’s psychomotor response to a single visual stimulus. Participants were instructed to fixate on a “+” symbol which was presented at the center of the screen for a variable amount of time (500 or 1000 ms). A 2 × 2 cm black square (target) was then presented in the middle of the screen and participants were instructed to respond with their right index finger on the spacebar, as fast as they can, when they detect the target. The target disappeared from the display after participant’s response or after 5000 ms. All 200 trials were organized in 4 blocks, 50 trials in each block. At the end of every block, the participant was granted a recess and was asked to initiate the next block by pressing any key on the keyboard.
2. **Choice Reaction Time (CRT):** In this task, the participants were asked to discriminate between two objects comprised from two different semantic categories. Each trial began with a ‘ + ’ symbol presented on the display for 350 ms followed by a picture of either “shirt” or “pants”. The stimuli were randomly selected from four different pictures of pants and four different pictures of shirts. The participants were asked to respond with either the ‘x’ key (left side on the keyboard) or the ‘m’ key (right side on the keyboard) to discriminate between the shirts and the pants. The assignment of keys (x/m) was counterbalanced between participants. The object disappeared from the display after participant’s response or after 5000 ms. A “beep” sound signaled error response. All objects were displayed 5 times, 40 trials over-all.
3. **Go/no-go:** Participants were required to fixate on a ‘ + ’ symbol which was presented at the center of the screen for a variable amount of time (300–1200 ms, leaps of 300 ms). Following fixating, the participants were presented with either the symbol ‘S’ or ‘$’ at the center of the display with a font-size equal to 40 pt. Participants were instructed to respond only when the ‘S’ symbol (target) appeared on the screen, by pressing the spacebar key with the right hand index finger as fast as they can. When the ‘$’ symbol (lure) appeared on the screen, participants were instructed to avoid responding. The presentation of the target or the lure symbols ended at the participant’s response or after 700 ms. A “beep” sound signaled error response. All 300 trials were organized in 6 blocks, 50 trials each. At the end of each block, the participant was granted a recess and was asked to initiate the next block by pressing any key on the keyboard.
4. **Anti-saccade:** This version of the task was adopted from Katzir, Eyal, Meiran, and Kessler [13]. Participants were instructed to fixate on a “+” symbol which was presented at the center of the screen for a variable amount of time (1500–3500 ms, leaps of 250 ms). A flashing black square (cue sign) was then flashed either to the left or right of fixation (distance from center 11.33° of visual angle). The interval between the cue (black square) and the target (an arrow) is called CTI (cue-target interval) and it varied between 200–500 ms (in leaps of 100 ms). The target (an arrow sign) appeared for 100 ms and then disappeared by a masking stimulus (black square) until a response was given. All targets appeared in the opposite location of the flashing cue. Participants had to indicate the direction of the target arrow (up, down, right or left) by pressing the corresponding arrow key on the keyboard. They performed first a practice block (8 trials) followed by 3 experimental similar blocks (44 trials each, 132 trials over-all).
5. **Dual task (Visual tracking** **+** **1-Back, Fig.** 1 **):** This version of the task was adopted from Cogscreen-AE battery [14]

  - **Visual tracking:** Participants were presented with a strait horizontal line (main line). The line extended to 80 % of the display width and positioned at 40 % of the display height. A small vertical line crossed the main line to mark the horizontal center of the display. Beneath the main line, a yellow triangle (target) was moving along the horizontal axis to either right or left. The pace of the moving target was fixed to 200 pixels per second. The participant was able to change the direction of the movement of the target by pressing the corresponding arrows keys using his right index finger. The instruction was to maintain the target at the center of the main line as long as possible. The task ended after 2 min. Figure 1a depicted the screen display of this task.
  - **1-Back:** On the same display as described above, the participants were asked to response according to the stimulus (number 1, 2 or 3) presented 1 trial backward. The number font-size was 82 pt at the center of the horizontal aspect of the display, 80 % of its height from the top. Each number was presented for 1000 ms followed by an inter-stimulus interval of 1000 ms. The participant’s task was to remember the previous number presented to him and press the correct number on the keyboard when the next number was presented. Figure 1b depicted the screen display of this task.
  - **Dual task**: In the Dual task condition, the participants were presented with both the visual tracking task and the 1-back task simultaneously. Figure 1c depicted the screen display of the dual task.
6. **Mental rotation test (MRT)**: This task is a modification of the original task by Shepard and Metzler [21]. The instructions presented on screen requested participants to decide whether pair of 3D shapes, each composed of 10 cubes, were identical (even if rotated) or different (See Fig. 2). Participants responded by pressing left (A) or right (L) on the keyboard. The assignment of keys to YES and NO responses was counterbalanced between participants. The pair of shapes was presented on screen until a response was made. Accuracy and response times in regards to the rotation angle measured. Each pair was presented to the right and to the left from the screen center with 7.6° visual angle between the shapes centers. The interval between the response and the next pair of shapes was 500 ms. Figure 2 depicted 2 pairs of shapes for demonstration.

### Procedure

The cognitive battery was installed on each participant’s personal laptop before the beginning of the experiment. 39 participants were randomly assigned to one of three groups (morning, late-afternoon, late evening). Each group performed a *single* session (60 min approximately) in one out of three different daytimes. We preferred to exhibit a single session for each participant in order to minimize the learning effect that occurs when repeating the same task more than once.

The single session protocol initiated with participants gathering in an isolated and quiet classroom at the IAF Flight Academy Campus. Each participant took his seat voluntarily and instructed first to close all running software, especially internet browsers, media software, anti-virus and all other resource consuming and visual pop-up software in order to prevent unwanted disturbances during the experiment. Then, each participant was instructed to open the main window of the cognitive battery on his personal laptop. Participants were instructed to sit straight in their chair, position the laptop screen approximately 60 cm in front of them with the screen center at the center of their visual field. It is important to note that all participants were using the laptops with the same configuration normally distributed to pilot-cadets by the IAF. During this initial phase, participants were also asked to complete a simple demographic questionnaire and were instructed to check their earphones and audio configuration.

After the initial phase, the participants performed all 6 cognitive tasks sequentially. For every task, a researcher orally explained the relevant instructions and participants were instructed to wait when completing each task until all the other participants completed their task in order to start the next task together with the rest of the group. This way, we ensured that all participants performed the tasks simultaneously. It also prevented participants’ motivation to perform fast at the expense of accuracy. After completion of all tasks, a research assistant collected the data from the personal laptops by means of a mobile memory device.

## Results

In order to assess the comparability of the groups, we conducted a series of one-way ANOVAs with Group (07A.M./06P.M./10P.M.) as the between-participant independent variable and task scores (SRT, CRT, Anti-saccade and Go/no-go) as the dependent variables. The analyses disclosed no statistically significant differences between groups in accuracy or reaction times in these measures (Table 1). However, in the MRT and in the dual task, we found interesting findings between the groups that will be presented here.

**Table 1:** Mean and standard deviation of SRT, CRT, Go-nogo, Anti-saccade

	SRT		CRT		Go-nogo		Anti-saccade	
	M	(SD)	M	(SD)	M	(SD)	M	(SD)
(a) Accuracy								
Morning group (N = 11)	1	*0*	*0.91*	*0.04*	0.95	*0.02*	0.82	*0.14*
Late- afternoon group (N = 13)	1	*0*	0.95	*0.03*	0.97	*0.02*	0.85	*0.05*
Late-evening group (N = 15)	1	*0*	0.94	*0.03*	0.96	*0.02*	0.827	*0.09*
Group main effect	ns		ns		ns		ns	
Morning group vs. late-afternoon group	ns		ns		ns		ns	
Late-afternoon vs. late-evening group	ns		ns		ns		ns	
Late-afternoon group vs. late-evening group	ns		ns		ns		ns	
(b) Reaction Time								
Morning group (N = 11)	238.07	*24.17*	*526.8*	*90.26*	466.2	*21.02*	504.69	*157.41*
Late-afternoon group (N = 13)	236.69	*20.49*	530.37	*61.88*	477.23	*18.44*	432.31	*77.82*
Late-evening group (N = 15)	234.73	*27.63*	492.8	*42.89*	471.38	*20.85*	447.72	*82.92*
Group main effect	ns		ns		ns		ns	
Morning group vs. late-afternoon group	ns		ns		ns		ns	
Late-afternoon vs. late-evening group	ns		ns		ns		ns	
Late-afternoon group vs. late-evening group	ns		ns		ns		ns	

### Mrt

**Fig. 3:**
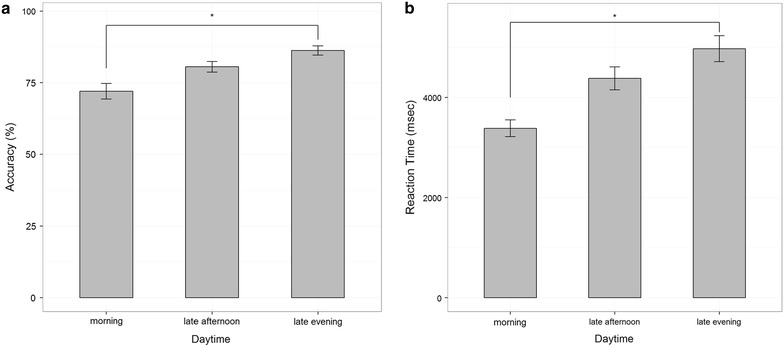
Accuracy and reaction time in the mental rotation task. **a** Differences between day time in accuracy (*Error bars* SE). **b** Daytime group diffrences in reaction time (*Error bars* SE)

Figure 3 depicts the averages of accuracy (Fig. 3a) and reaction times (Fig. 3b) by Group (07:00 a.m./06:00 p.m./10:00 p.m.). Accuracy and RT were compared by means of ANOVAs between the three groups. The analysis indicated a significant effect of ‘time of day’ on the participants’ accuracy [*F* (2, 32) = 3.4, *p* < 0.05]. In a post hoc pairwise t tests with Bonferroni alpha correction for multiple comparisons, we found a near significant (*p* = 0.52) increase in participants’ accuracy (*M* = 0.14, *SD* = 0.05) between the morning group and the late evening group. The differences between participant’s accuracy at the morning and late-afternoon groups were found insignificant. The differences between participant’s accuracy at the late evening and late-afternoon groups were also found insignificant (Fig. 3a).

The analysis also indicated a significant effect of ‘time of day’ on the participants’ RT [*F* (2, 32) = 4.5, *p* < 0.05]. In a post hoc pairwise t tests with Bonferroni alpha correction for multiple comparisons, we found a significant increase (*p* < 0.05) in participants’ RT from morning to late evening group (*M* = 1571.68, *SD* = 509.31). The differences between participant’s RT at morning and late-afternoon groups were found insignificant. The differences between participant’s RT at the late evening and late-afternoon groups were also found insignificant (Fig. 3b).

### Dual task

The Dual task was analyzed by comparing the participant’s performance on the 1-back task and the visual tracking task when performed separately to the performance of each task as performed in the dual task condition.

**Fig. 4:**
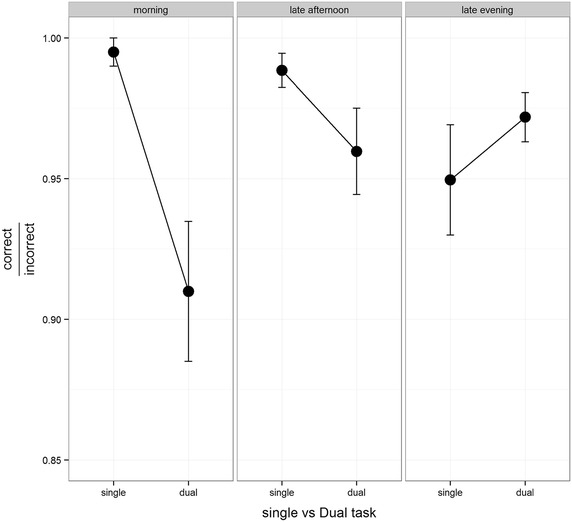
Percentage of correct responses in the 1-back task. The *right column* represent the morning group single vs. dual task (*Error bars* SE). The *middle column* represent the results of the late afternoon group (*Error bars* SE) and the *left column* represent the results of the late night group (*Error bars* SE)

In the 1-back task, participants were required to respond accurately to the previous number presented on the screen. Therefore, mean accuracy was defined as the proportion of the correct responses in all 20 trials of 1-back task. A mixed design ANOVA analysis was performed in order to test the influence of ‘time of day’ and dual tasking on accuracy in the 1-back task. The dual task factor was considered as a within subject with two levels (single task, dual task) and the ‘time of day’ as a between subject with three levels (morning, late-afternoon, late evening). Figure 4 depicted the accuracy differences in the 1-back task between single and dual-tasking conditions in the three different daytimes. In an ANOVA analysis for interaction effects, we found a main effect for dual tasking [*F* (1, 33) = 5.4*, p* < 0.05] meaning that the accuracy in the 1-back task on the single task condition was significantly higher than on the dual task condition. We also found a differential effect of dual tasking on accuracy in the different daytimes [*F* (2, 33) = 5.6, *p* < 0.01]. In a post hoc analysis with Bonferroni correction for multiple comparisons, we found that accuracy in the 1-back task deteriorates from single task condition to the dual task condition only in the morning group (p < 0.05), but not in the late evening or late-afternoon group (Fig. 4).

For the analysis of the visual tracking, we calculated the time that the participant expended at the center of the display. The center of the display was defined as 10 pixels extended to the right and left from the display center. This measure is attributed as the centser-time (CT). Analysis of the effect of ‘time of day’ and dual tasking on CT followed a mixed design model. The dual tasking factor considered as a within subject with two levels (single task, dual task) and the ‘time of day’ as a between subject with three levels (morning, late-afternoon, late evening). In an ANOVA analysis for interaction effects, we found no ‘time of day’ effect on CT, when the task performed in a single task condition or in a dual task condition [*F*_(2, 31)_ = 0.12, *p* = *ns*].

## Discussion

Aviators require high cognitive control abilities. They are operating in very high mental-load scenarios and a decline in their cognitive control abilities may have a crucial negative effect on their flying performance. Consequently, the cadet population is more vulnerable because they are young and unexperienced but are still expected to maintain high level of performance for prolonged hours during training days.

Our findings suggest that in this highly functioning population, there are no differences in performance on simple tasks that involve low-mental load (SRT, go/no-go task, anti-saccade and CRT) for the different hours of the day. However, in tasks that are high-mental load consuming, we found a difference in performance for the different hours of the day. In the MRT, which is a task that involves visual WM [11, 15], we found very impulsive performance- fast and not accurate- in the morning group (07:00 a.m.), and more precise yet slower performance in the late evening group (10:00 p.m.). We suggest that this ‘trade-off’ behavior in performance on the MRT in the late evening group (slowing down in order to perform better) may be a result of a voluntary control mechanism (top-down process) activated at night, in this group. The combination of feeling fatigue, along with the understanding that this specific task is more resource consuming (the cadets reported that the MRT was the most difficult task in the cognitive battery) caused them to check and double-check before pressing the button and answer, whereas in the morning group, they felt alert and vital, and acted more reactively ended in impulsive manner and caused inaccurate performance.

Similar results were also found in the dual task, a task known to be a resource consuming activity [1, 12]. In this study, we found a decrement in performance (accuracy scores) on the 1-Back task during the dual task condition for the morning group but not for the late evening group, a finding that is in line with the MRT results. Once again, the morning group behaved in an impulsive way and showed a decrease in accuracy, while in the late evening group, participants were probably aware of their lowered cognitive capacity at night and therefore activated compensating cognitive control functions in order to overcome their fatigue. It is also reasonable that the differential effect in the dual task was found only in the 1-back task and not in the visual tracking since it is known that the motor reaction is more automatic and strong than working memory (executive function ability).

In a future study, we suggest performing the same procedure again and adding another morning group that is lectured regarding the influence of cognitive control mechanisms on performance *before* performing the cognitive battery. Our hypothesis is that after awareness, the results of this morning group will be more similar to that of the late evening group due to the activation of voluntary cognitive control abilities.

## Limitations

The use of one test session for each pilot-cadet in this study prevented influence of practice/learning effect that is inevitable as a result of test-retest examination. It is possible that the differential results found were due to individual differences. However, it is less likely to assume, since we found consistent results in both complex tasks. It a future study it will also be interesting to add a group of non-pilot population preforming the same test-battery. Another limitation is the relatively small sample size, therefore a replication study should be conducted so as to increase validity. Lastly, the sleeping habits of the young cadets were not thoroughly investigated in this study, which may have affected our results. Future research can examine this possibility through the use of subjective questionnaires such as the Morning–Evening Questionnaire (MEQ; [10]) or the Munich Chrono-type Questionnaire (MCTQ; [20]).

## Footnotes

1 The reason for unequal group numbers was previous engagements of some of the cadet pilots in the day of the experiment.

## References

[1] Bratzke D, Rolke B, Ulrich R, Peters M (2007). Central slowing during the night. Psychol Sci.

[2] Braver TS (2012). The variable nature of cognitive control: a dual mechanisms framework. Trends Cogn Sci.

[3] Caldwell JA, Caldwell JL, Smythe NK, Hall KK (2000). A double-blind, placebo-controlled investigation of the efficacy of modafinil for sustaining the alertness and performance of aviators: a helicopter simulator study. Psychopharmacology.

[4] Caldwell J, Caldwell L, Smith J, Alvarado L, Heintz T. The efficacy of modafinil for sustaining alertness and simulator flight performance in F-117 pilots during 37 hours of continuous wakefulness (No. AFRL-HE-BR-TR-2004-0003). AIR FORCE RESEARCH LAB BROOKS AFB TX HUMAN EFFECTIVENESS DIR/BIODYNAMICS ANDPROTECTION DIV; 2004.

[5] Caldwell JA, Caldwell JL, Brown DL, Smith JK (2004). The effects of 37 hours without sleep on the performance of F-117 pilots. Mil Psychol.

[6] Caldwell JA, Mu Q, Smith JK (2005). Are individual differences in fatigue vulnerability related to baseline differences in cortical activity?. Behav Neurosci.

[7] Caldwell JA, Mallis MM, Caldwell JL, Paul MA, Miller JC, Neri DF (2009). Fatigue countermeasures in aviation. Aviat Space Environ Med.

[8] Chandler JF, Arnold RD, Phillips JB, Turnmire AE (2013). Predicting individual differences in response to sleep loss: application of current techniques. Aviat Space Environ Med.

[9] Folkard S, Åkerstedt T. Towards a model for the prediction of alertness and/or fatigue on different sleep/wake schedules. Contemporary advances in shiftwork research. Krakow: Medical Academy. 1987:231–240.‏.

[10] Horne JA, Ostberg O (1975). A self-assessment questionnaire to determine morningness– eveningness in human circadian rhythms. Int J Chronobiol.

[11] Hyun JS, Luck SJ (2007). Visual working memory as the substrate for mental rotation. Psychon Bull Rev.

[12] Jasper I, Roenneberg T, Häußler A, Zierdt A, Marquardt C, Hermsdörfer J (2010). Circadian rhythm in force tracking and in dual task costs. Chronobiol Int.

[13] Katzir M, Eyal T, Meiran N, Kessler Y (2010). Imagined positive emotions and inhibitory control: the differentiated effect of pride versus happiness. J Exp Psychol Learn Mem Cogn.

[14] Kay GG (1995). Cogscreen Aeromedical Edition Professional Manual.

[15] Kaufman SB (2007). Sex differences in mental rotation and spatial visualization ability: can they be accuracy ounted for by differences in working memory capacity. Intelligence.

[16] Killgore WDS, Grugle NL, Reichardt RM, Killgore DB, Balkin TJ (2009). Executive functions and the ability to sustain vigilance during sleep loss. Aviat Space Environ Med.

[17] Mathôt S, Schreij D, Theeuwes J (2012). OpenSesame: an open-source, graphical experiment builder for the social sciences. Behavior Res Methods.

[18] McCauley P, Kalachev LV, Smith AD, Belenky G, Dinges DF, Van Dongen H (2009). A new mathematical model for the homeostatic effects of sleep loss on neurobehavioral performance. J Theor Biol.

[19] McClelland LE, Pilcher JJ, Moore DD (2010). Oculomotor Measures as Predictors of Performance during Sleep Deprivation. Aviat Space Environ Med.

[20] Roenneberg T, Wirz-Justice A, Merrow M (2003). Life between clocks: daily temporal patterns of human chronotypes. J Biol Rhythms.

[21] Shepard RN, Metzler J (1971). Mental Rotation of Three-Dimensional Objects. Science.

[22] Spencer MB (1987). The influence of irregularity of rest and activity on performance: a model based on time since sleep and time of day. Ergonomics.

[23] Van Dongen HP (2005). Brain activation patterns and individual differences in working memory impairment during sleep deprivation. Sleep.

[24] Van Dongen HP, Caldwell JA, Caldwell JL (2006). Investigating systematic individual differences in sleep-deprived performance on a high-fidelity flight simulator. Behavior Res Methods.

